# Tandem Catalysis as a Platform for the Mild Synthesis of Renewable Aliphatic Polyanhydrides

**DOI:** 10.1002/cssc.71206

**Published:** 2026-11-05

**Authors:** Lucie Fournier, Jakhongir Bekmirzaev, Malaury Simon, Fanny Betermier, Carine Robert, Robert T. Mathers, Régis M. Gauvin, Christophe M. Thomas

**Affiliations:** ^1^ Chimie ParisTech, PSL University, CNRS Institut de Recherche de Chimie Paris Paris France; ^2^ Department of Chemistry Pennsylvania State University New Kensington Pennsylvania USA

**Keywords:** olefin metathesis, polyanhydrides, renewable feedstocks, step‐growth polymerization, tandem catalysis

## Abstract

The development of one‐pot catalytic strategies that transform renewable feedstocks into functional polymers remains an important challenge in sustainable polymer chemistry. Here, we report a tandem‐catalyzed strategy for the mild step‐growth polymerization of a fatty acid‐derived monomer into high‐molar‐mass aliphatic polyanhydrides. Selective ruthenium‐catalyzed self‐metathesis of 10‐undecenoic acid affords eicos‐10‐enedioic acid with high conversion and minimal olefin positional isomerization within the substrate. The resulting diacid is subsequently converted into a polyanhydride through magnesium‐catalyzed dehydrative polycondensation using dimethyl dicarbonate as a stoichiometric dehydrating reagent. The two catalytic transformations are operationally compatible and can be performed as a sequential one‐pot process without isolation of the diacid intermediate. The orthogonal Ru/Mg sequence affords polyanhydrides with weight‐average molar masses above 30 kg/mol, while the independently optimized magnesium‐catalyzed polymerization reaches weight‐average molar masses above 80 kg/mol. A ruthenium species derived from the metathesis catalyst can also promote the dehydrative coupling step, providing an auto‐tandem pathway, although with lower molar‐mass control. The resulting polyanhydride films exhibit water contact angles of 95°–100°, within the range reported for polyethylene and polypropylene surfaces. This work establishes a mild and step‐efficient route from a renewable unsaturated fatty acid to hydrophobic aliphatic polyanhydrides.

## Graphical abstract

Tandem catalysis enables the one‐pot conversion of renewable 10‐undecenoic acid into high‐molar‐mass aliphatic polyanhydrides under mild conditions. Selective ruthenium‐catalyzed self‐metathesis followed by magnesium‐catalyzed dehydrative polycondensation avoids isolation of the diacid intermediate and affords hydrophobic, semicrystalline materials, establishing a step‐efficient platform for sustainable polyanhydride synthesis.

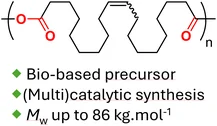

## 1 Introduction

Aliphatic polyanhydrides constitute a distinctive class of biodegradable polymers characterized by their surface‐erosion behavior, hydrophobicity, and biocompatibility [1, 2]. In contrast to polyesters or polyamides, hydrophobic polyanhydrides limit water penetration into the bulk, resulting in linear erosion kinetics and predictable mass loss while maintaining the mechanical integrity of the material throughout most of the degradation process [3, 4, 5, 6, 7, 8, 9, 10]. Despite these highly advantageous properties and the relative chemical simplicity of condensation polymerization, the broader use of polyanhydrides has remained limited by the harsh and poorly sustainable conditions traditionally required for their synthesis. Conventional routes rely on toxic activating agents and high‐temperature melt polycondensation [11, 12, 13]. These approaches not only raise sustainability and safety concerns but also complicate processing, restrict functional group tolerance, and often lead to polymers with limited molar mass control and broad dispersity [14]. This apparent paradox has hampered the development of sustainable and modular access to polyanhydrides, particularly from renewable feedstocks. A general catalytic approach that enables the direct conversion of dicarboxylic acids into high‐molar‐mass polyanhydrides under mild conditions has yet to be realized.

In this context, one‐pot catalysis provides a powerful conceptual framework to address this gap by enabling the sequential activation and transformation of functional groups within a single catalytic sequence [15]. Such approaches offer several decisive advantages over stepwise methodologies: they eliminate the need for intermediate isolation, reduce solvent use, and improve safety by minimizing the accumulation of reactive intermediates [16]. For polymer synthesis, one‐pot catalysis is particularly attractive because it can provide direct access to macromolecules from simple feedstocks while improving step economy, limiting intermediate handling, and potentially reducing solvent and energy requirements. Although one‐pot catalysis has been widely exploited in small‐molecule synthesis, its application to polymer synthesis, particularly to step‐growth processes involving sensitive functional groups, remains comparatively underdeveloped and would represent a significant advance toward more sustainable polymer synthesis [17, 18, 19, 20].

Herein, we report a tandem‐catalyzed strategy for the sequential one‐pot synthesis of an aliphatic polyanhydride from a renewable fatty acid‐derived precursor (Scheme 1). The approach builds on 10‐undecenoic (undecylenic) acid, an industrially relevant, castor‐oil‐derived ω‐unsaturated fatty acid with a terminal C═C bond and a key platform intermediate for value chains such as Nylon‐11 [21]. This work thus provides a mild and step‐efficient alternative to conventional polyanhydride syntheses that rely on more forcing activation procedures or high‐temperature melt polycondensation.

**SCHEME 1:**
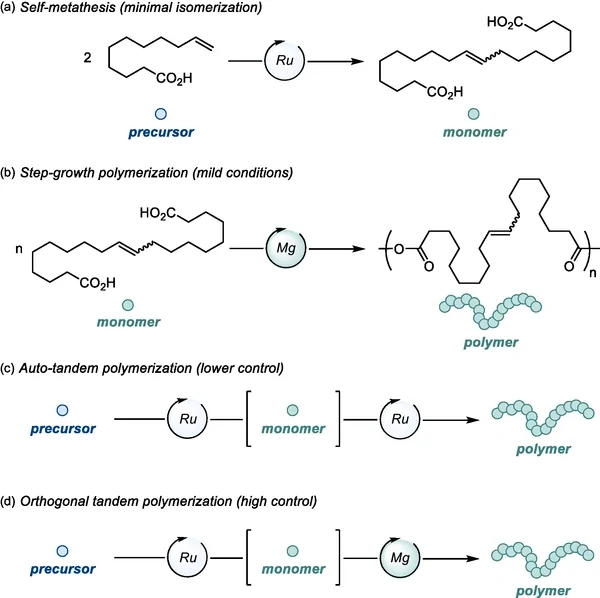
(a) Self‐metathesis of 10‐undecenoic acid. (b) Polycondensation of eicos‐10‐enedioic acid. (c) Auto‐tandem polymerization of 10‐undecenoic acid. (d) Orthogonal tandem polymerization of 10‐undecenoic acid.

## 2 Results and Discussion

### 2.1 Design of the Tandem Catalytic Strategy

To enable an efficient one‐pot process, the self‐metathesis of undecenoic acid must proceed with both high conversion and minimal olefin positional isomerization, as even small amounts of monoacid byproducts compromise access to high‐molar‐mass polymers. However, ruthenium metathesis catalysts are well known to promote double‐bond migration under forcing conditions, particularly with terminal alkenes [22, 23, 24, 25, 26, 27].

### 2.2 Selective Access to the Diacid Monomer

Guided by the need for simultaneous high conversion and minimal olefin positional isomerization, several commercially available Ru catalysts tolerant to polar and protic functionalities were therefore evaluated under neat conditions, using continuous ethylene removal to drive the equilibrium (Figure 1, Table 1). While the M1 catalyst and Zhan‐1C showed moderate activity (Table 1, entries 1 and 2), significant isomerization was observed at high conversion (see the Supporting Information). In contrast, Zhan‐1B provided near‐quantitative conversion (97%) within 3 h with minimal isomerization, outperforming the other systems tested (Table 1, entry 3). Although the supported Zhan‐1B formulation could in principle facilitate catalyst separation and reuse, its lower activity and greater propensity for positional isomerization precluded meaningful recycling studies under the present conditions (Table 1, entry 4). Given its superior performance, Zhan‐1B was selected for further optimization. The marked differences between these different types of olefin metathesis catalysts are in line with the established balance between activity and stability, with lack of stability being connected to the isomerization side reaction [28, 29].

**FIGURE 1:**
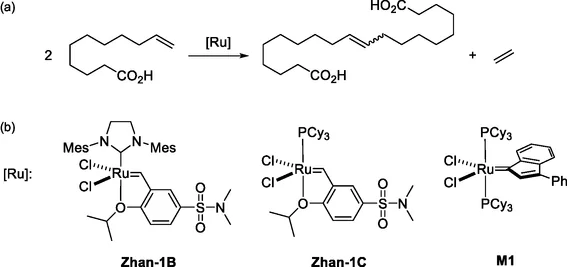
(a) Self‐metathesis of 10‐undecenoic acid and (b) ruthenium catalysts used in this study.

**TABLE 1:** Self‐metathesis of undecenoic acid with different catalysts.^a^

Entry	Catalyst	Time, h	Conv., %^b^	Self‐metathesis selectivity, %^b^	Isomerization, %^b^
1	M1	3	63	62	38
		22	95	25	75
2	Zhan‐1C	3	36	86	14
		22	91	59	41
3	Zhan‐1B	3	97	92	8
4	Supported Zhan‐1B	3	42	65	35
		22	96	36	64

a 200 equiv. of acid, 110 °C, 100 mbar.

b Determined by ^1^H NMR spectroscopy.

To suppress residual olefin migration, additives known to inhibit double‐bond isomerization were examined [30]. Under solvent‐free conditions, conversion remained high, but incomplete suppression of isomerization was observed. Switching to dimethyl carbonate (DMC), a green and metathesis‐compatible solvent, improved reaction homogeneity and reproducibility, affording promising catalytic performances (Table 2, entry 1) [31]. Among the additives evaluated, acetic acid proved to be particularly effective. Using 5 equivalents of acetic acid relative to ruthenium, complete conversion was achieved within 30 min without detectable olefin isomerization under the optimized screening conditions (Table 2, entry 4).

**TABLE 2:** Self‐metathesis of 10‐undecenoic acid with different additives.^a^

Entry	Additive	[Additive]/[Ru]	Time, h	Conv., %^b^	Self‐metathesis selectivity, %^b^	Isom., %^b^
1	—	—	0.5	>99	81	9
2	1,4‐benzoquinone	2	23	95	94	6
3	1,4‐benzoquinone	5	28	93	>99	0
4	Acetic acid	5	0.5	>99^c^	>99	0

a Zhan‐1B as catalyst, 500 equiv. of 10‐undecenoic acid at 1 M in DMC, reflux, atmospheric pressure.

b Determined by ^1^H NMR spectroscopy.

c Eicos‐10‐enedioic acid was isolated as a white solid in 35% yield after removal of DMC, dissolution of the residue in dichloromethane, and precipitation into cold pentane. The difference between NMR selectivity and isolated yield primarily reflects material losses during purification.

Notably, the beneficial effect of acetic acid is observed despite the large excess of carboxylic acid groups present in the substrate, suggesting a specific interaction with the positional isomerization pathway rather than a general acidic effect. Overall, the combination of Zhan‐1B and acetic acid in DMC provides a highly selective route to eicos‐10‐enedioic acid. The diacid can be obtained as a white solid after removal of DMC, dissolution in dichloromethane, and precipitation into cold pentane in moderate isolated yield. The difference between the high NMR‐determined conversion and the moderate isolated yield primarily reflects material losses during purification rather than extensive formation of unidentified soluble byproducts. Importantly, the crude diacid mixture is of sufficient quality for direct use in the subsequent polycondensation step, thereby avoiding the substantial material losses associated with isolation and purification of the intermediate. This observation suggested the possibility of merging monomer synthesis and polymer formation into a single catalytic sequence. We thus explored the feasibility of a one‐pot approach combining olefin metathesis and dehydrative polycondensation in one reaction vessel.

### 2.3 Catalytic Polycondensation Under Mild Conditions

With a diacid monomer available under mild and catalytic conditions, we next turned our attention to its conversion into polyanhydrides. A central objective was to identify a catalytic polycondensation strategy capable of operating under solution conditions, thereby avoiding the harsh activation methods and high temperatures typically required for anhydride formation. Building on recent advances in metal‐mediated dehydrative coupling, we next explored a magnesium‐catalyzed polycondensation strategy compatible with the diacid obtained from metathesis [32, 33, 34, 35]. Magnesium salts have been shown to promote the cyclization of dicarboxylic acids into cyclic anhydrides in the presence of dicarbonate reagents, suggesting that intermolecular coupling could be favored when cyclization is disfavored by ring size [36]. In the case of eicos‐10‐enedioic acid, intramolecular anhydride formation is thermodynamically unlikely, opening the possibility of step‐growth polymerization under appropriately mild conditions. Preliminary experiments using MgCl_2_ confirmed its ability to promote anhydride bond formation at low temperature; however, the heterogeneous nature of this system resulted in limited reproducibility and control (Table S1). We therefore turned to the well‐defined and commercially available magnesium amide complex (TMP)MgCl·LiCl (TMP = 2,2,6,6‐tetramethylpiperidyl, Figure 2) [38], whose enhanced solubility and reactivity are well documented. This catalyst was expected to generate reactive magnesium carboxylate species capable of activating dicarbonate coupling agents while maintaining compatibility with solution‐phase polymerization [39, 40, 41, 42, 43, 44, 45].

**FIGURE 2:**
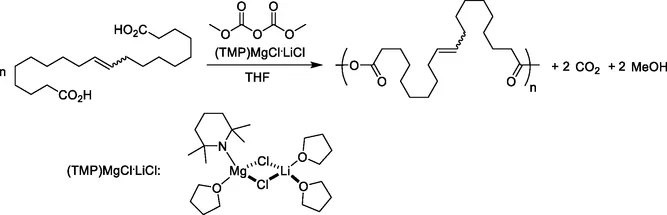
Eicos‐10‐enedioic acid polymerization and structure of THF‐solvated (TMP)MgCl·LiCl [37].

Using dimethyl dicarbonate as a stoichiometric activating and dehydrating reagent operating under mild conditions, we systematically investigated the solution polycondensation of eicos‐10‐enedioic acid as a function of monomer concentration, catalyst loading, and dehydrating‐agent stoichiometry. Under standard conditions (0.1 M, [Moc_2_O]/[diacid] = 1.0), the reaction afforded polyanhydride with a weight‐average molar mass (*M*_w_) of 14,820 g mol^−1^ after 1 h (Table 3, entry 1). Increasing the amount of Moc_2_O to 1.25 equiv. led to higher conversion and a marked increase in *M*_w_ (Table 3, entry 2), reaching 33,390 g mol^−1^ within 30 min. A slight excess of Moc_2_O proved beneficial, consistent with the near‐quantitative functional‐group conversion required to achieve high molar masses in step‐growth polymerization. We propose that part of the activating reagent may be consumed through nonproductive pathways, including methanol‐mediated esterification and chain capping. Increasing the Moc_2_O loading from 1.0 to 1.25 equiv. may compensate for these losses and thereby increase both conversion and molar mass. This interpretation remains a working hypothesis based on the observed dependence on reagent stoichiometry. Di‐*tert*‐butyl dicarbonate was also evaluated as an alternative dehydrating reagent. Under the conditions optimized for dimethyl dicarbonate, Boc_2_O showed substantially lower reactivity, with no conversion after 1 h at 0.1 M. At higher concentration and after prolonged reaction, however, conversion above 99% was achieved, affording a polymer with an apparent *M*_w_ of 18.4 kg/mol after 20 h. Thus, Boc_2_O is competent but considerably slower and less effective than Moc_2_O for rapid access to high‐molar‐mass polyanhydrides (Table S2). Although dimethyl dicarbonate enables anhydride formation under mild conditions, its 1.25‐fold stoichiometric excess contributes appreciably to the material footprint of the polymerization. The environmental benefits associated with the low reaction temperature, renewable monomer, and DMC solvent must therefore be weighed against the production, handling, and consumption of this reactive activating reagent. At this stage, the transformation is best considered a mild, step‐efficient proof of concept, with further progress requiring reduced reagent loading, recovery strategies, or catalytic alternatives.

**TABLE 3:** Magnesium‐catalyzed polycondensation of eicos‐10‐enedioic acid.^a^

Entry	[Moc_2_O]/[diacid]	[diacid], M	[diacid]/[Mg]	Time, h	Conv.^b^, %	*M*_w_ c, g mol^−1^	*Ð* c
1	1	0.1	10,000	1	82	14,820	1.9
2	1.25	0.1	10,000	0.5	>99	33,390	2.1
3	1.25	1	10,000	0.25	96	33,660	2.2
4	1.25	0.1	100	1	89	16,910	1.9
5	1.25	0.1	1000	0.5	>99	86,250	2.3

a General conditions: reactions in THF at 40 °C using (TMP)MgCl·LiCl as the catalyst.

b Conversion of carboxylic acid functions into anhydride linkages, as determined by ^1^H NMR spectroscopy.

c *M*_w_ and *Ð* were determined by SEC‐RI in THF at 35 °C using polystyrene standards.

The influence of concentration was subsequently assessed at fixed catalyst loading and [Moc_2_O]/[diacid] = 1.25. At higher monomer concentration (1.0 M), reduced reaction times resulted in lower average molar masses and incomplete conversion (Table 3, entry 3), consistent with diminished selectivity under these conditions. Catalyst loading also had a pronounced effect. A high magnesium loading ([diacid]/[Mg] = 100) promoted rapid polymer formation but afforded only moderate conversion and molar mass (Table 3, entry 4). In contrast, a lower catalyst loading corresponding to [diacid]/[Mg] = 1000 produced the highest molar masses, with *M*_w_ values of up to 86,250 g mol^−1^ (Table 3, entry 5). These results indicate that excessive catalyst loading may increase the concentration of initiating or chain‐capping species and thereby limit molar‐mass build‐up. Preliminary MALDI‐TOF analyses of polymers obtained during the early stages of this study revealed distributions consistent with the presence of cyclic species, suggesting that intramolecular cyclization may provide an additional pathway affecting molar‐mass build‐up. Although analogous analyses could not be obtained for the optimized high‐molar‐mass samples, cyclic products may also form under the present solution‐polycondensation conditions. Their contribution to the molar‐mass distributions observed by SEC remains to be established. Notably, the resulting polymer exhibited good solution stability in CDCl_3_, showing no detectable change by ^1^H NMR spectroscopy over 1 week and retaining 88% of its anhydride linkages after 1 month, indicating encouraging resistance toward hydrolytic degradation under these storage conditions.

### 2.4 Orthogonal Tandem Catalysis: One‐Pot Synthesis of Polyanhydrides

To implement an efficient orthogonal tandem sequence, we evaluated whether the ruthenium metathesis catalyst (Zhan‐1B) and the magnesium polycondensation catalyst ((TMP)MgCl·LiCl) could operate sequentially in a single vessel without mutual deactivation. A key constraint is that the metathesis step must deliver the diacid intermediate with minimal olefin positional isomerization since even small amounts of monoacid impurities are expected to cap chains and limit molar‐mass build‐up in step‐growth polymerization (Figure 3).

**FIGURE 3:**
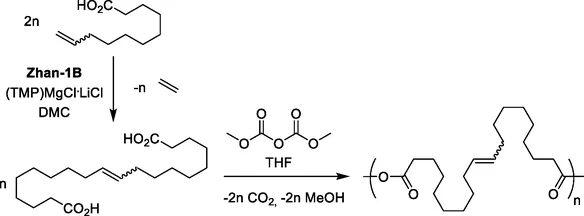
Orthogonal tandem catalysis for poly(eicos‐10‐ene‐1,20‐dicarboxylic anhydride) synthesis.

Accordingly, the orthogonal tandem sequence was initiated by self‐metathesis of 10‐undecenoic acid in DMC using Zhan‐1B in the presence of acetic acid (5 equiv. relative to Ru) and a catalytic amount of (TMP)MgCl·LiCl (0.025 equiv.). Under these conditions, complete consumption of 10‐undecenoic acid was achieved within 30 min, with no detectable positional olefin isomerization. This result indicates that the magnesium complex does not measurably interfere with the metathesis step under these conditions. After completion of the metathesis step, DMC was removed under reduced pressure without isolation of the diacid intermediate. The residue was redissolved in THF and dimethyl dicarbonate (1.25 equiv. relative to the diacid) was added under an inert atmosphere. After stirring at 40 °C for 1 h, the orthogonal tandem sequence afforded the target polyanhydride with a weight‐average molar mass of 30,800 g mol^−1^ and a dispersity (*Đ*) of 2.0, in excellent agreement with the values observed under analogous conditions in the standalone polycondensation experiments. These results collectively demonstrate that ruthenium‐catalyzed metathesis and magnesium‐mediated polycondensation are operationally compatible and can be concatenated in a one‐pot, orthogonal tandem sequence without intermediate purification. This preserves step economy while maintaining polymerization efficiency and molar‐mass control.

### 2.5 Auto‐Tandem Catalysis: One Catalyst, Two Transformations

Having established an orthogonal Ru/Mg tandem manifold, we next investigated whether the metathesis catalyst itself could mediate both steps, thereby enabling an auto‐tandem pathway in which a ruthenium catalyst precursor introduced for the metathesis step also gives rise to species capable of promoting the subsequent step‐growth polycondensation (Figure 4). We reasoned that, beyond olefin metathesis, ruthenium (carbene) complexes can display Lewis‐acidic and/or electrophilic behavior and have been reported to promote a range of nonmetathetic transformations under appropriate conditions [46, 47, 48, 49, 50, 51, 52, 53, 54, 55, 56, 57, 58, 59, 60, 61, 62, 63].

**FIGURE 4:**
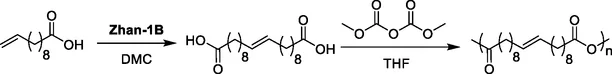
Auto‐tandem catalysis for poly(eicos‐10‐ene‐1,20‐dicarboxylic anhydride) synthesis.

To probe this possibility, the self‐metathesis of 10‐undecenoic acid (500 equiv. relative to Ru) was first conducted in refluxing DMC using Zhan‐1B as the sole metal catalyst in the presence of acetic acid as an additive. Full consumption of the starting acid was achieved within 30 min. After completion of the metathesis step, DMC was removed under reduced pressure, and the residue was redissolved in THF (1.0 M). Dimethyl dicarbonate (1.25 equiv.) was then added under an inert atmosphere, and the reaction mixture was stirred at 40 °C for 2 h, affording a polyanhydride with a weight‐average molar mass of 11,840 g mol^−1^ and a dispersity of 1.6. Notably, gel permeation chromatography revealed the emergence of a secondary distribution, indicating diminished control relative to the magnesium‐catalyzed polycondensation (vide supra) and suggesting the formation of lower‐molar‐mass species and/or side products under the auto‐tandem regime (Figure S9). Consistent with a control experiment in which an independently prepared polyanhydride was exposed to Zhan‐1B under conditions representative of the second step, no major change in its SEC distribution was observed. This result suggests that secondary metathesis of the internal backbone olefins does not measurably alter the molar‐mass distribution under these conditions. Control experiments confirmed that no significant polymer formation occurs in the absence of a catalyst, supporting that the observed activity is attributable to the ruthenium complex rather than to background reactivity of Moc_2_O. Importantly, these results show that a ruthenium species derived from Zhan‐1B can promote both the metathesis and dehydrative coupling stages, thereby providing an auto‐tandem entry to the polyanhydride. Because the identity of the ruthenium species responsible for the second transformation has not been established, the result is interpreted as a proof of concept rather than as evidence that the intact ruthenium carbene complex catalyzes both reactions. The auto‐tandem pathway also provides lower molar mass and poorer distribution control than the orthogonal Ru/Mg sequence.

### 2.6 Physico‐Chemical Properties

The thermal and structural properties of a freshly prepared high molar mass polyanhydride film (*M*_w_ = 63.2 kg mol^−1^) were assessed by thermogravimetric analysis, differential scanning calorimetry (DSC), and X‐ray diffraction. The polymer displayed a temperature at 5% mass loss (*T*_d,−5%_) of 233 °C under nitrogen (Figure S10). DSC analysis revealed a melting transition at ≈57 °C and a crystallization event at ≈42 °C (Figure S11). The X‐ray diffraction pattern was similar to that reported for poly(sebacic anhydride), supporting the formation of a semicrystalline long‐chain aliphatic polyanhydride (Figure S12) [64]. Interestingly, the diffraction pattern differed from those commonly reported for related long‐chain aliphatic polyesters, suggesting that the anhydride linkages influence chain packing [65]. A detailed assignment of the crystalline structure is, however, beyond the scope of the present study.

To explore the scope of potential applications, the hydrophobicity of the eicos‐10‐enedioic acid‐derived polyanhydrides was investigated with complementary experimental and computational techniques and compared with a variety of known polymers. First, the films displayed static water contact angles of 95.2° ± 3.0° (average of eight measures) and 99.9° ± 3.8° (average of six measures) for polymers with *M*_w_ values of 23,000 and 45,000 g mol^−1^, respectively. Second, macromolecular hydrophobicity parameters (MHP) were calculated by constructing a series of oligomeric molecular models of the polyanhydride in Figure 3 [66, 67]. Then, computational octanol‐water partition coefficients (Log*P*) were calculated (Equation (1)) and normalized by the surface area of the molecular model (SA). The resulting Log*P*/SA values provide a predictive metric to quantify hydrophobicity [68, 69].

(1) $$\mathrm{Log}P=\log\left(\frac{\left[{\text{solute}}_{\text{octanol}}\right]}{\left[{\text{solute}}_{\text{water}}\right]}\right)$$

As shown in Figure 5, the experimental contact angles and calculated MHP indicate that the polyanhydride surfaces are more hydrophobic than several common polymers containing degradable or polar functional groups (e.g., Nylon 6, poly(ε‐caprolactone), and poly(ethylene terephthalate)) and compared well with the range of values reported for polyethylene (PE) (94° [70], 104.4° [71]) and polypropylene (PP) (95° [72], 104° [66, 67]) films. For comparison, contact angles for the eicos‐10‐enedioic acid‐derived polyanhydrides are slightly higher than the range of reported values for polyanhydrides using thiol‐ene chemistry (82°–92° [73]) and salicylic acid‐derived poly(anhydride‐esters) (75°–95° [74]) groups.

**FIGURE 5:**
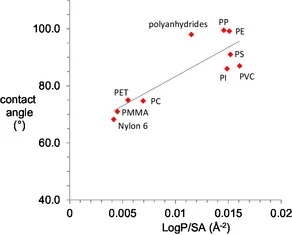
Comparison of water contact angles and macromolecular hydrophobicity parameters (MHP) for polyanhydrides in Figure 3 with Nylon 6, polymethylmethacrylate (PMMA), poly(ε‐caprolactone) (PC), poly(ethylene terephthalate) (PET), polyisoprene (PI), polyvinylchloride (PVC), polystyrene (PS), polyethylene (PE), and polypropylene (PP).

## 3 Conclusion

In summary, we have developed a mild tandem‐catalyzed strategy for converting renewable 10‐undecenoic acid into a long‐chain aliphatic polyanhydride. Selective ruthenium‐catalyzed self‐metathesis affords eicos‐10‐enedioic acid with high conversion and minimal olefin positional isomerization, while magnesium‐catalyzed dehydrative polycondensation using dimethyl dicarbonate provides high‐molar‐mass polyanhydrides at 40 °C.

The main outcome of this work is the operational compatibility of the ruthenium and magnesium catalysts. This compatibility enables a sequential one‐pot, two‐step process in which the diacid intermediate is used directly without isolation or purification. The orthogonal Ru/Mg sequence therefore improves step economy and avoids the material losses associated with isolation of the intermediate. A ruthenium species derived from Zhan‐1B can also promote the second transformation, providing an auto‐tandem pathway, although with lower molar‐mass and poorer control over molar‐mass distribution.

The resulting semicrystalline polyanhydride combines a renewable long‐chain aliphatic backbone with thermally labile anhydride linkages and exhibits a hydrophobic surface, with water contact angles within the range reported for PE and PP. More broadly, the results demonstrate that tandem catalysis can connect renewable monomer synthesis and step‐growth polymerization under mild conditions. The present system provides a proof of concept for applying this strategy to the preparation of other anhydride‐containing polymers from unsaturated carboxylic‐acid feedstocks.

## Conflicts of Interest

The authors declare no conflicts of interest.

## Supporting information

Supplementary Material

## Data Availability

The data that support the findings of this study are available from the corresponding author upon reasonable request.
