# Supplementary material for: Tandem Catalysis as a Platform for the Mild Synthesis of Renewable Aliphatic Polyanhydrides
Source: ChemSusChem. 2026 Nov 5;19(21):e71206. doi: 10.1002/cssc.71206 (PMC13738052; doi:10.1002/cssc.71206)
Supplement: Supplementary file 1 [file CSSC-19-e71206-s001.pdf]

Tandem Catalysis as a Platform for the Mild Synthesis of Renewable Aliphatic Polyanhydrides

Lucie Fournier,[a] Jakhongir Bekmirzaev,[a] Malaury Simon,[a] Fanny Betermier,[a] Carine Robert,[a] Robert T. Mathers,[b] Régis M. Gauvin[a] and Christophe M. Thomas*[a]

[a] Chimie ParisTech, PSL University, CNRS, Institut de Recherche de Chimie Paris, 75005 Paris, France
[b] Department of Chemistry, Pennsylvania State University, New Kensington, Pennsylvania 15068, USA
*Corresponding author: christophe.thomas@chimie-paristech.fr

Table of Contents

General considerations S2
Representative eicos-10-enedioic acid synthesis with benzoquinone as an additive S4
Representative eicos-10-enedioic acid synthesis with acetic acid as an additive S4
Large scale synthesis of eicos-10-enedioic acid S5
Figure S1. ^{1}H NMR spectrum of eicos-10-enedioic acid S5
Figure S2. ^{13}C{^{1}H} NMR spectrum of eicos-10-enedioic acid S6
Figure S3. IR spectrum of eicos-10-enedioic acid S6
Figure S4. ^{1}H NMR spectrum of undecenoic acid metathesis with high isomerization rate S7
Table S1. Investigations on undecenoic anhydride formation S7
Representative synthesis of poly(eicos-10-ene-1,20-dicarboxylic anhydride) S7
Figure S5. ^{1}H NMR spectrum of poly(eicos-10-ene-1,20-dicarboxylic anhydride) S8
Figure S6. ^{13}C{^{1}H} NMR spectrum of poly(eicos-10-ene-1,20-dicarboxylic anhydride) S9
Figure S7. IR spectrum of poly(eicos-10-ene-1,20-dicarboxylic anhydride) S9
Table S2. Polycondensation of eicos-10-enedioic acid using different dialkyldicarbonate reagents. S10
Table S3. Polycondensation of eicos-10-enedioic acid using different solvents S10
Orthogonal tandem synthesis of poly(eicos-10-ene-1,20-dicarboxylic anhydride) S10
Figure S8. SEC chromatogram of the polyanhydride obtained by orthogonal tandem catalysis S11
Auto-tandem synthesis of poly(eicos-10-ene-1,20-dicarboxylic anhydride) S11
Figure S9. SEC chromatogram of the polyanhydride obtained by auto-tandem catalysis S12
Formation of and investigations on a representative polyanhydride film S12
Figure S10. TGA trace of polyanhydride S13
Figure S11. DSC trace of polyanhydride S13
Figure S12. X-ray diffractogram of polyanhydride and sample holder S14
Figure S13. Side view of a water droplet on polyanhydride S14

General considerations.

All manipulations requiring a dry atmosphere were performed under a purified argon atmosphere using standard Schlenk techniques or in a glovebox. THF, DMF and diethyl ether were dried through a drying columns purification system from Innovative Technologies, or dried on the appropriate agent, then degassed by freeze–thaw–vacuum cycles prior to use. Catalytic runs were prepared using a Jacomex GP [concept] glovebox equipped with a O_2 and H_2O purification system. ^1H and ^{13}C-{^1H} NMR spectra were recorded on Bruker Avance300, a Bruker Avance400 or a Bruker Avance500 spectrometers at 297 K at 300 MHz and 75 MHz, 400 MHz and 100 MHz or 500 MHz and 125 MHz, respectively and referenced to the residual solvent peaks. Size exclusion chromatography (SEC) was performed in THF at 35 °C using an Agilent 1260 Infinity Series GPC (ResiPore 3 µm, 300 ×7.5 mm, 1.0 mL min-1, UV (250 nm) and RI (PLGPC 220) detectors. The weight average molar masses (M_w) and dispersities ($Đ$) of the polymers were determined by SEC using polystyrene standards for calibration (limits M_w = 200 to 400 000 g mol^{-1}). The thermal stability was evaluated using a thermogravimetric analyzer (TGA55, TA Instruments). Samples (1.5 ± 0.5 mg) were heated from room temperature to 400 °C on a platinum pan at a heating rate of 10 °C/min under nitrogen flow rate of 25 mL/min. $T_{d,-5\%}$ of polymers was defined as the thermal degradation temperature corresponding to 5% weight loss. Melting and crystallization temperature were determined using a differential scanning calorimeter (DSC25, TA Instrument) under a nitrogen flow of 25 mL/min. Samples (1.5 ± 0.5 mg) were sealed in aluminum pans with pierced lid. To erase thermal history of polymer, a first heat/cool/heat cycle from -50 °C to 200 °C was used. As a representative run, the first heating was done from -50 °C to 200 °C at 10 °C/min; then the cooling was done from 200 °C to -50 °C at -5 °C/min; the final heating was done from -50 °C to 200 °C at 10 °C/min. X-ray diffraction was carried out on a Panalytical MRD diffractometer with a copper anode as a source and acquisition in the range 2ϑ = [10-70] °. Diffuse reflectance Fourier transform infrared

measurements were carried out on a Thermo Fisher Nicolet IS 20 FT-IR spectrometer equipped with a Harrick Praying Mantis device.

Dimethyl dicarbonate (purchased from Aldrich) was put through three freeze-pump-thaw cycles and stored in the freezer inside the glovebox. Undecenoic acid was purchased from Alfa Aesar and degassed by freeze–thaw–vacuum cycles prior to use. Anhydrous $MgCl_2$ (purchased from Sigma-Aldrich) was used as received. Di-*tert*-butyl dicarbonate (purchased from Fluorochem) was stored in the freezer and used as received. 2,2,6,6-Tetramethylpiperidinylmagnesium chloride lithium chloride (purchased from Sigma-Aldrich) was stored in the freezer in the glovebox. 1,3-Bis(2,4,6-trimethylphenyl)-4,5-dihydroimidazol-2-ylidene[2-(*i*-propoxy)-5-(*N*,*N*-dimethylaminosulfonyl)phenyl]methylene ruthenium (II) dichloride (Zhan-1B catalyst) and {[2-(*i*-propoxy)-5-(*N*,*N*-dimethylaminosulfonyl)phenyl]methylene}(tricyclohexylphos-phine) ruthenium(II) dichloride (Zhan-1C catalyst) were purchased from Strem Chemicals and stored in a glovebox. Dichloro(3-phenyl-1H-inden-1-ylidene)bis(tricyclohexylphosphine) ruthenium(II) (M1 catalyst) was purchased from Umicore and stored in a glovebox. 1,4-benzoquinone was purchased from Aldrich and sublimated prior to use. Acetic acid was purchased from Alfa Aesar, distilled over acetic anhydride and $KMnO_4$ and stored under argon. 2-methyltetrahydrofuran was purchased for Alfa Aesar, dried over sodium, put through three freeze-pump-thaw cycles, then vacuum transferred under argon (twice) and stored in a glove box. Dimethyl carbonate was purchased from Acros Organics, dried over CaH_2, and distilled prior use. For the conversion of undecenoic acid by olefin metathesis, conversion and selectivity towards self-metathesis and isomerization were calculated by ^{1}H NMR as follows:

Conversion (%)= $100\times[1-(I_{CH\text{-}UA})/(I_{CH\text{-}UA} + 2\times I_{CH\text{-}DA})]$

Selectivity to self-metathesis product (%)=$100\times[(I_{CH\text{-}DA}-(\Sigma\ I_{Me}/3))/(I_{CH\text{-}DA})]$

Isomerization (%)= $100\times[(\Sigma\ I_{Me})/(3\times I_{CH\text{-}DA})]$

With $I_{CH\text{-}UA}$: integration of a single olefinic proton from undecenoic acid, $I_{CH\text{-}DA}$: integration of a single olefinic proton from eicos-10-enedioic acid, i.e. half the integration of the 5.37 ppm peak, and $\Sigma\ I_{Me}$ the sum of the integration of the methyl peaks (triplets resonating below 1.0 ppm).

Representative eicos-10-enedioic acid synthesis with benzoquinone as an additive

In a glovebox, 10-undecenoic acid (0.500 g, 2.72 mmol, 500 eq.), 1,4-benzoquinone (1.2 mg, 10.8 μmol, 2 eq.) and Zhan-1B (4.0 mg, 5.4 μmol, 1 eq.) were placed in a Schlenk tube. The tube was removed from the glovebox and heated at 100°C under dynamic 100 mbar vacuum. After 2 hours, the reaction mixture was cooled at room temperature. ^{1}H NMR analysis of the crude mixture indicated 96% conversion of 10-undecenoic acid. The crude mixture was then dissolved in dichloromethane and precipitated in cold pentane (ice bath) to yield the desired product as a white solid (0.319 g, 69 % isolated yield).

Representative eicos-10-enedioic acid synthesis with acetic acid as an additive

In a glovebox, 10-undecenoic acid (965 mg, 5.25 mmol, 500 eq.), acetic acid (3 μL, 52.5 μmol, 5 eq.), Zhan-1B (7.7 mg, 10.5 μmol, 1 eq.) and dimethyl carbonate (5.3 mL) were placed in a Schlenk tube. The Schlenk tube was removed from the glovebox, connected to a Schlenk line and the reaction mixture was heated at reflux for 30 minutes. ^{1}H NMR analysis of the crude mixture indicated full conversion of 10-undecenoic acid with no detectable positional olefin isomerization. The solvent was removed under reduced pressure, and the crude residue was dissolved in dichloromethane and precipitated into cold pentane. Eicos-10-enedioic acid was isolated as a white solid (315 mg, 35% isolated yield).

^{1}H NMR (400 MHz, $CDCl_3$, δ ppm): 5.37 (m, 2H, =C*H*), 2.35 (m, 4H, C*H₂*COOH), 1.97 (m, 4H, =CHC*H₂*), 1.64 (m, 4H, C*H₂*CH₂COOH), 1.30 (m, 10H, =CHCH₂(C*H₂*)₅(CH₂)₂COOH).

^{13}C NMR (100 MHz, $CDCl_3$, δ ppm): 180.4 (*C*OOH), 130.5 (=*C*H), 34.2 (*C*H₂COOH), 32.7 (=CH*C*H₂(CH₂)₇COOH), 29.3 (=CHCH₂(*C*H₂)₅(CH₂)₂COOH), 24.8 (*C*H₂CH₂COOH).

Large scale synthesis of eicos-10-enedioic acid

In a glovebox, 10-undecenoic acid (32.3 g, 176 mmol, 1,000 eq.), 1,4-benzoquinone (38 mg, 0.35 mmol, 2 eq.) and Zhan-1B (129 mg, 0.176 mmol, 1 eq.) were placed in a Schlenk tube. The tube was removed from the glovebox and heated at 100°C under a dynamic pressure of 300 mbar for 2 h. After cooling to room temperature, the crude reaction mixture was precipitated into pentane. Eicos-10-enedioic acid was isolated as a white solid (12 g, 40 % isolated yield). ICP analysis indicated a residual ruthenium content of 5.8 $\mu mol.g^{-1}$.

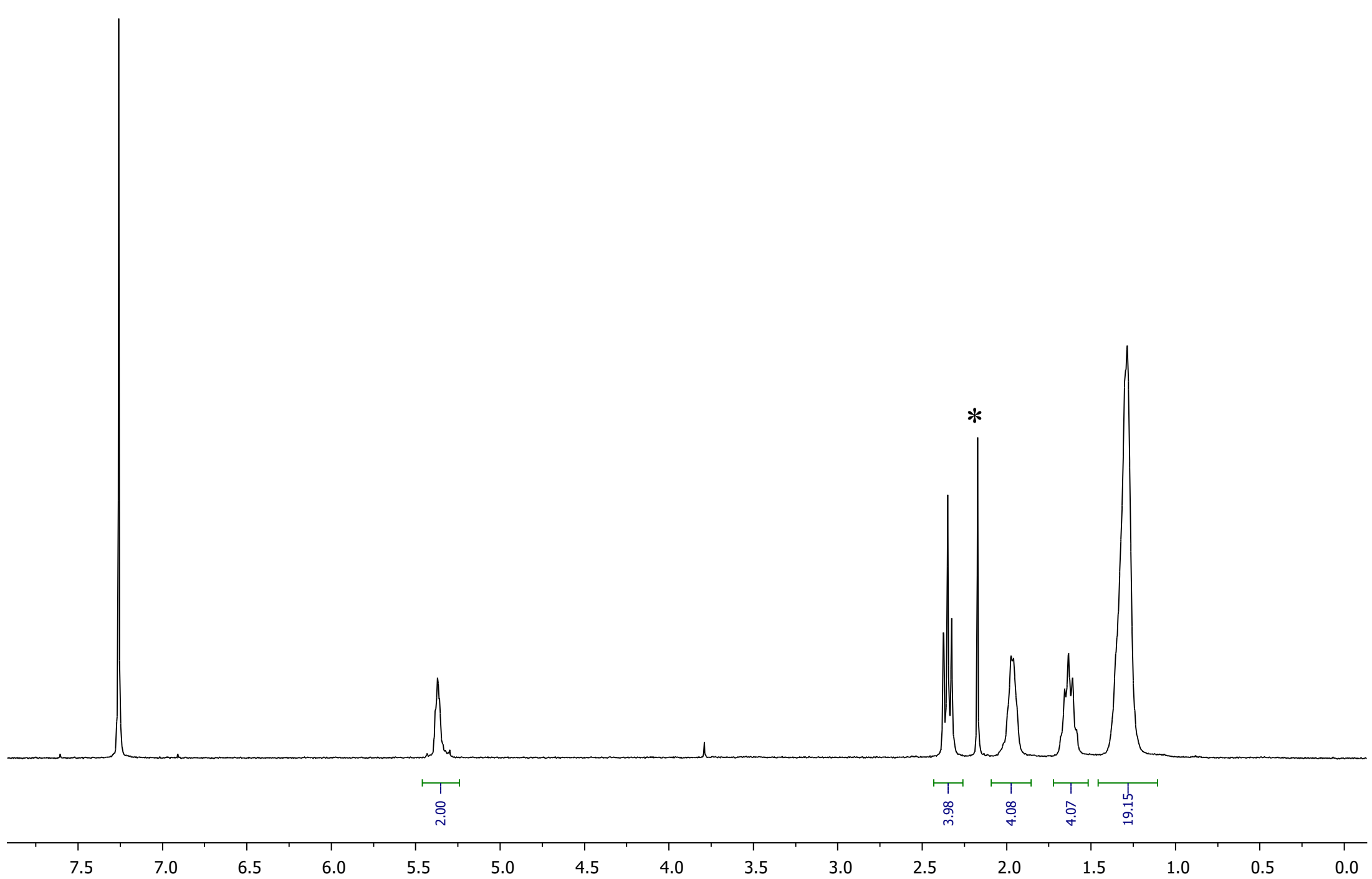

Figure S1: ^{1}H NMR spectrum of eicos-10-enedioic acid (400 MHz, $CDCl_3$)

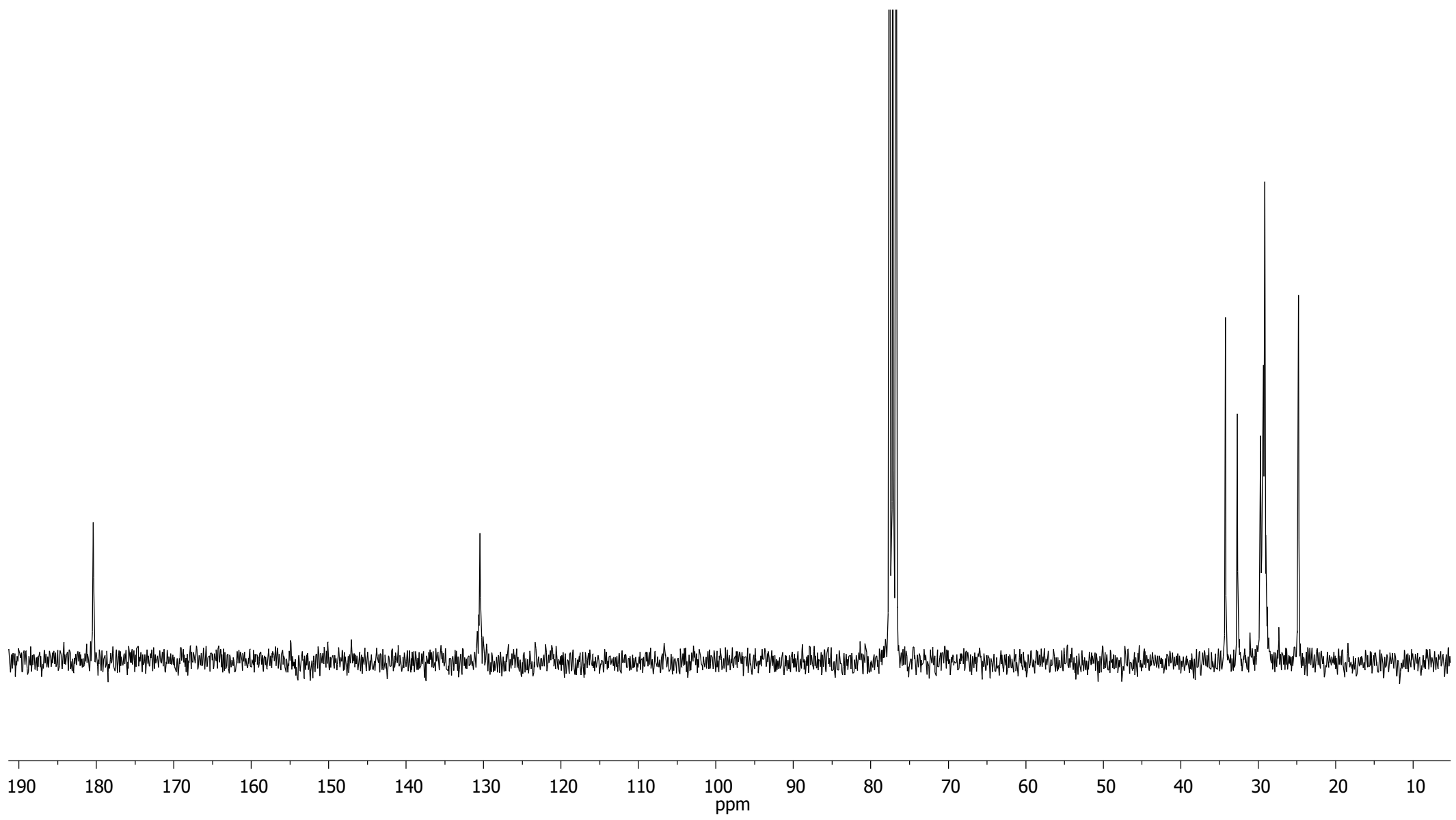

Figure S2. $^{13}C\{^{1}H\}$ **NMR spectrum of eicos-10-enedioic acid (75 MHz,** $CDCl_3$**)**

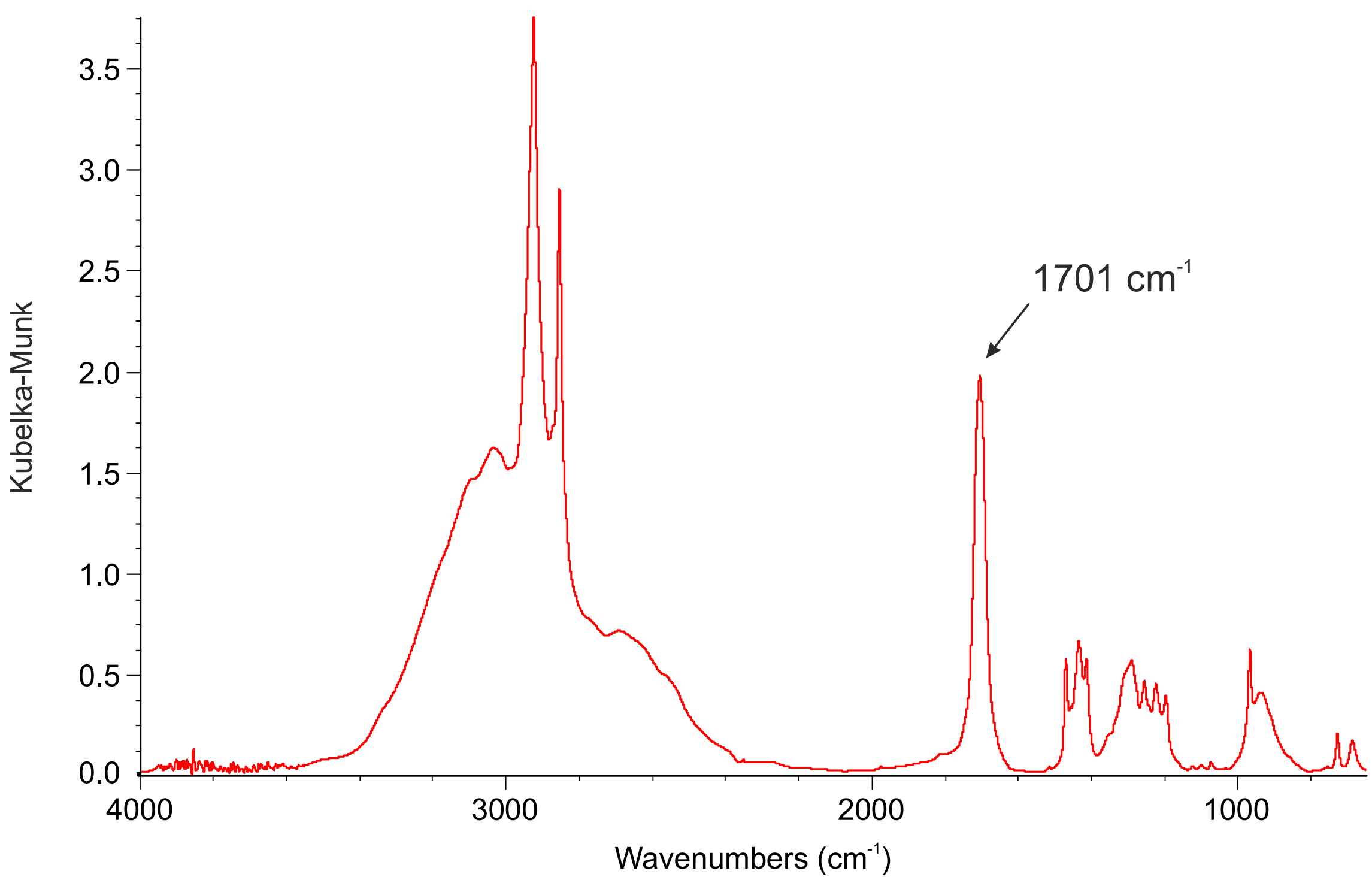

Figure S3. IR spectrum of eicos-10-enedioic acid

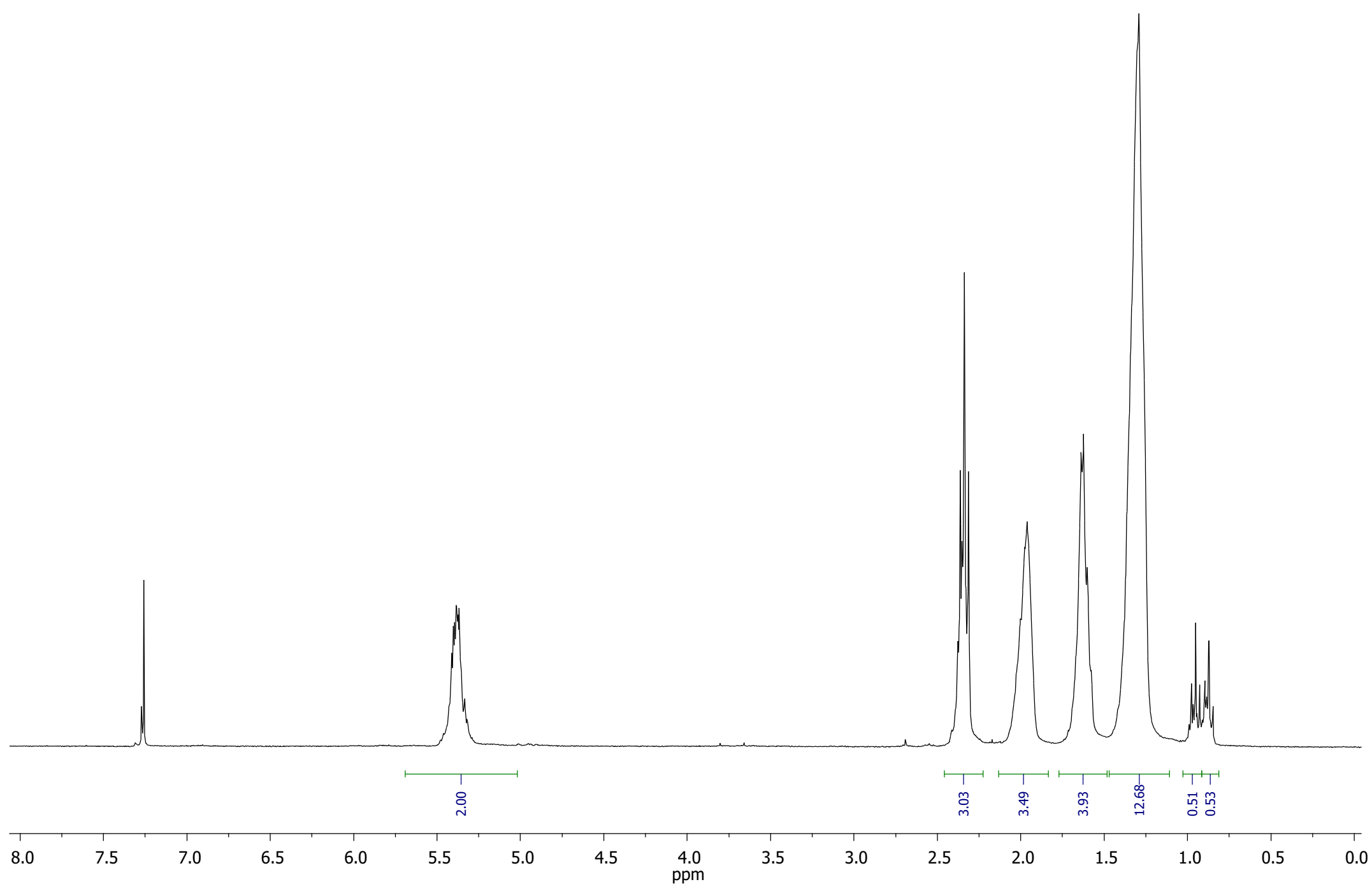

Figure S4. ^{1}H NMR spectrum of a representative example of undecenoic acid metathesis with high isomerization rate (400 MHz, $CDCl_3$)

Table S1. Control experiments for the dehydrative coupling of 10-undecenoic acid.[a]

Entry	Catalyst	Conversion[b] (%)
**1**	TMPMgCl•LiCl	> 99
**2**	/	0
**3**	TMPH	2
**4**	LiCl	0
**5**	$MgCl_2$	54

[a] 10-undecenoic acid (1 M in THF, 1000 eq.), Boc_2O (600 eq.), catalyst (1 eq.), 2 h, 40 °C. [b] Determined by ^{1}H NMR spectroscopy.

Representative synthesis of poly(eicos-10-ene-1,20-dicarboxylic anhydride)

In a glovebox, eicos-10-enedioic acid (350 mg, 1.02 mmol, 10,000 eq.), and a solution of (TMP)MgCl·LiCl in THF (10 µL of a 0.010 M solution, 0.10 µmol, 1 eq.) were placed in a

Schlenk tube. The tube was removed from the glovebox, and THF (10 mL) and dimethyl dicarbonate (0.14 mL, 1.275 mmol, 1,25 eq. relative to the diacid) were added under argon. The reaction mixture was stirred at 40 °C for the specified time. The solvent and volatile by-products were then removed under reduced pressure. The crude polymer was dissolved in dichloromethane and passed through a short plug of silica. Removal of the solvent under reduced pressure afforded poly(eicos-10-ene-1,20-dicarboxylic anhydride) as a white solid.

^{1}H NMR (400 MHz, $CDCl_3$, δ ppm): 5.37 (m, 2H, =CH), 2.43 (m, 4H, C$\mathit{H_2}$COOCO), 1.96 (m, 4H, =CHC$\mathit{H_2}$), 1.65 (m, 4H, C$\mathit{H_2}$$CH_2$CO), 1.27 (m, 10H, =CH$CH_2$(C$\mathit{H_2}$)$_5$($CH_2$)$_2$CO).

^{13}C NMR (100 MHz, $CDCl_3$, δ ppm): 169.8 (COOCO), 130.5 (=CH), 35.4 ($\mathit{C}$$H_2$COOCO), 32.7 (=CH$\mathit{C}$$H_2$), 29.7-28.5 (=CH$CH_2$($\mathit{C}$$H_2$)$_5$($CH_2$)$_2$CO), 24.3 ($\mathit{C}$$H_2$$CH_2$CO).

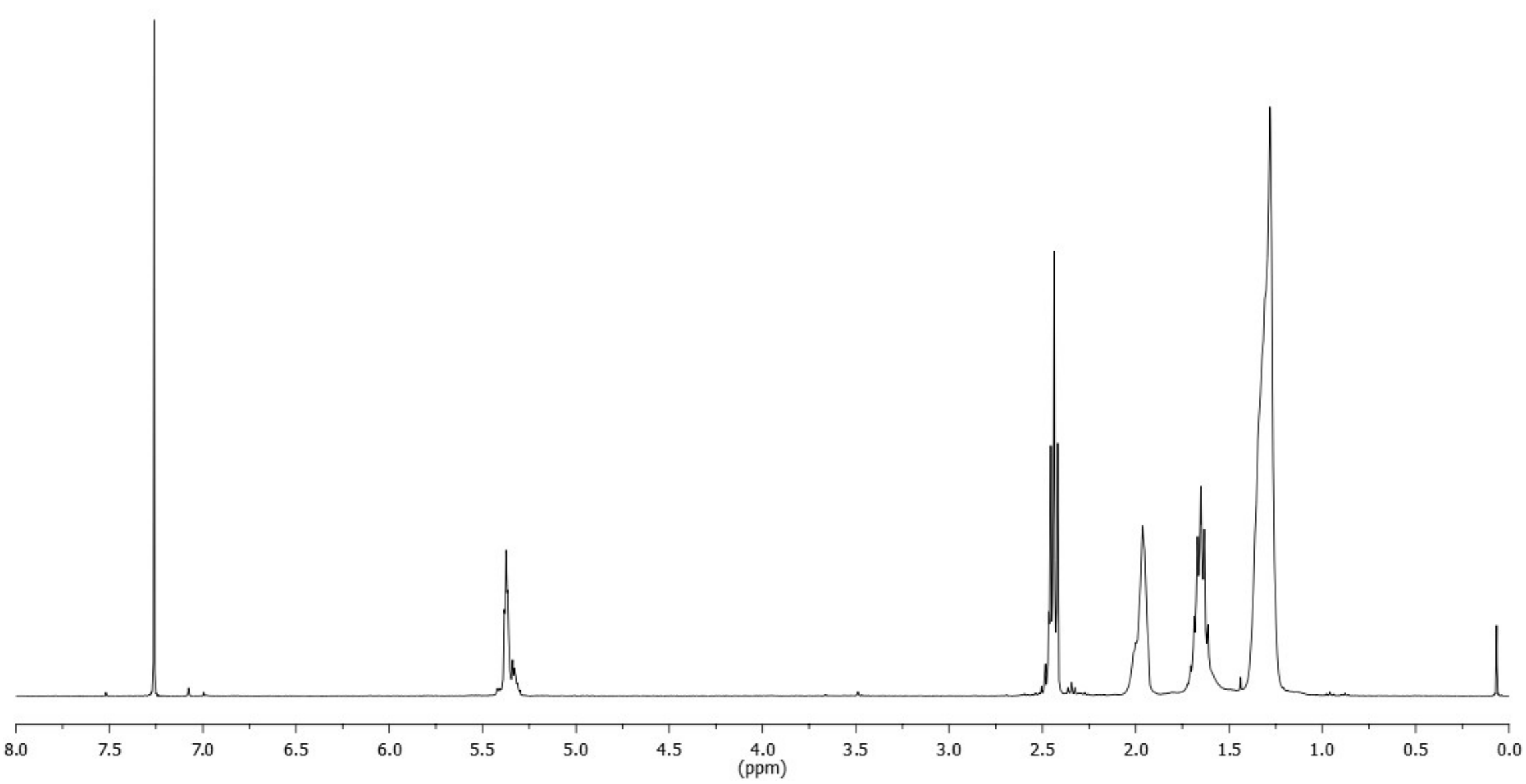

Figure S5. ^{1}H NMR spectrum of poly(eicos-10-ene-1,20-dicarboxylic anhydride) (400 MHz, $CDCl_3$)

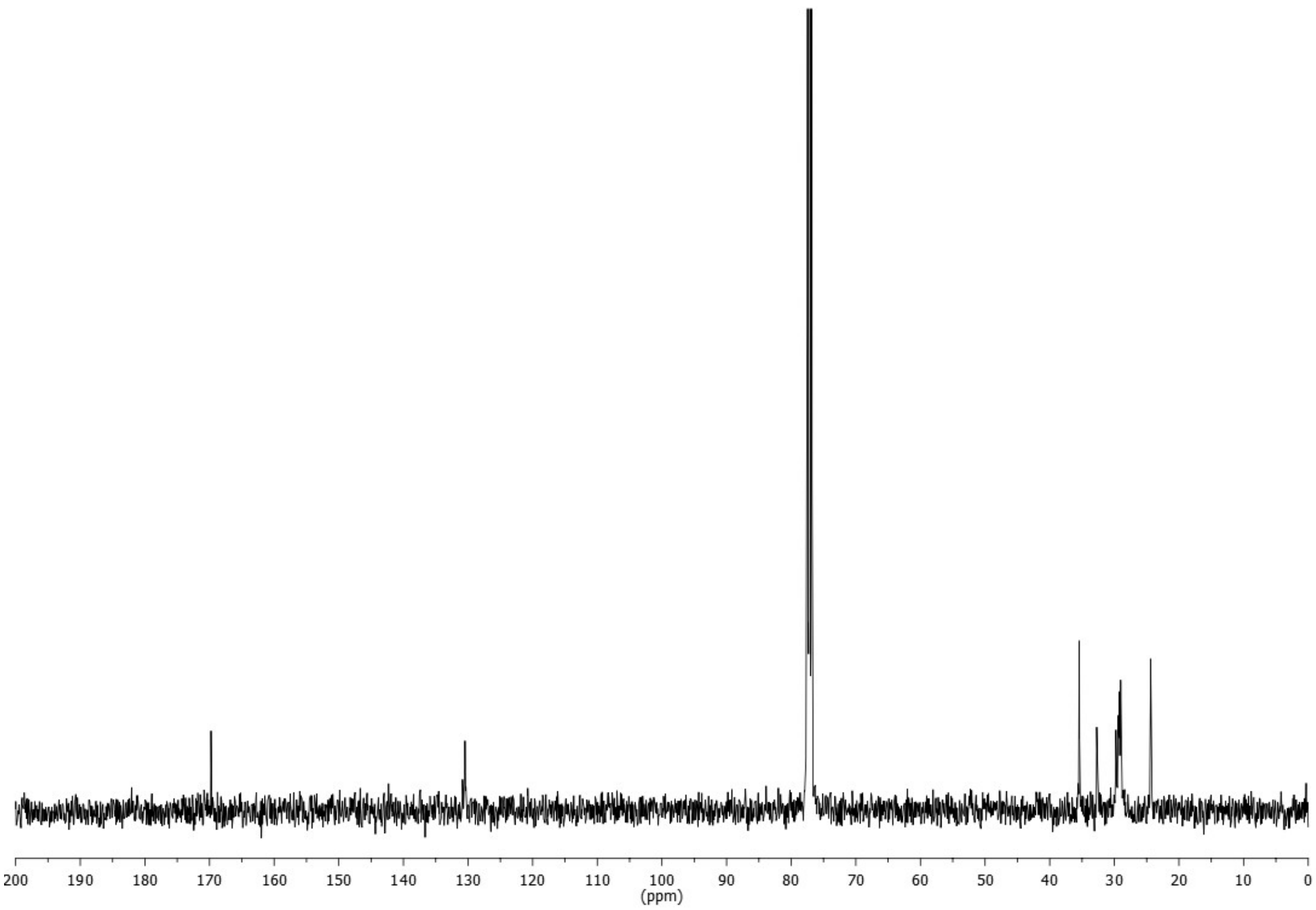

Figure S6. $^{13}C\{^1H\}$ NMR spectrum of poly(eicos-10-ene-1,20-dicarboxylic anhydride) (100 MHz, $CDCl_3$)

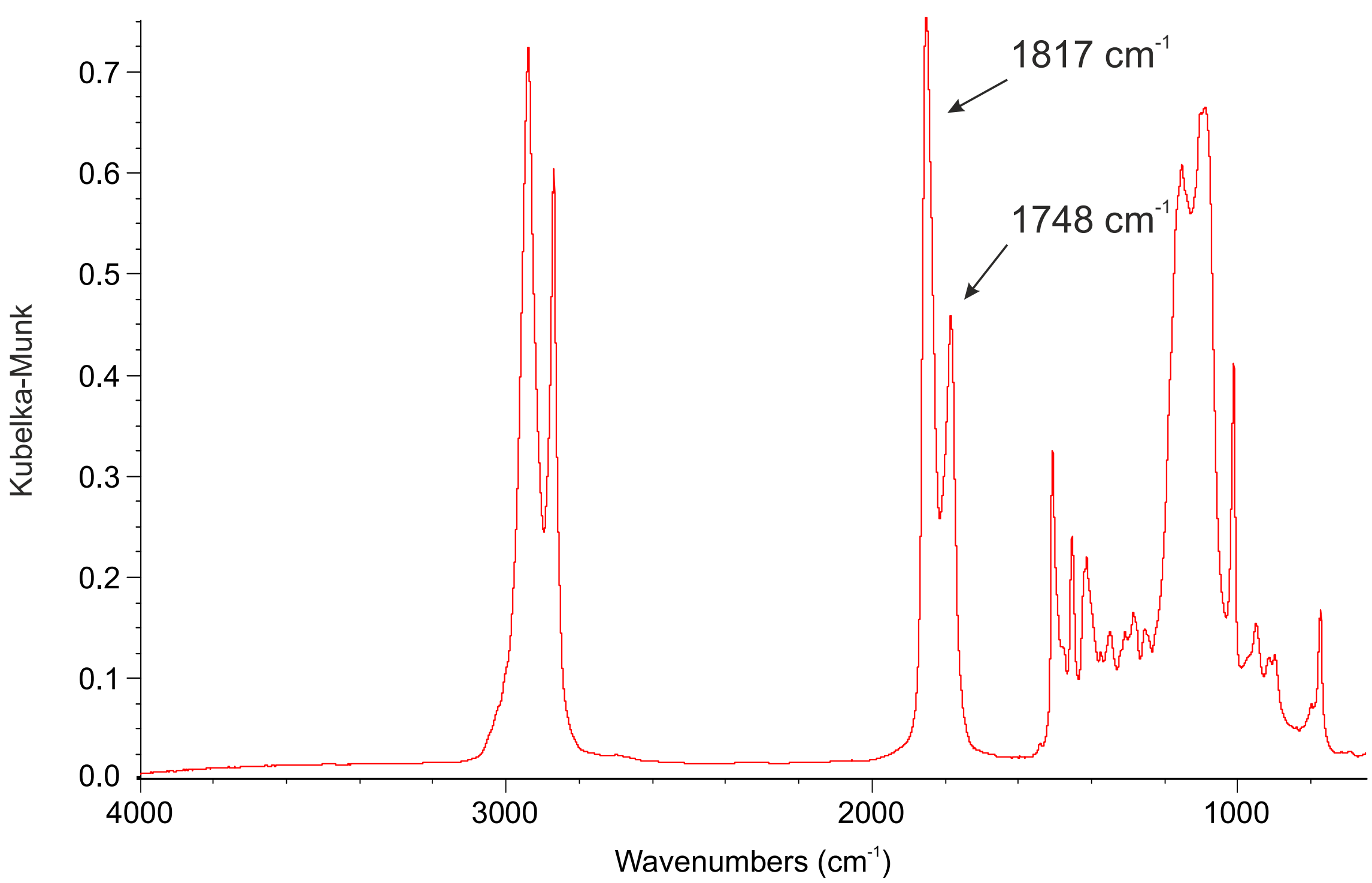

Figure S7. IR spectrum of poly(eicos-10-ene-1,20-dicarboxylic anhydride)

Table S2. Influence of the dicarbonate reagent on the magnesium-catalyzed polycondensation of eicos-10-enedioic acid[a]

Entry	Dicarbonate	Conc. (M)	Time (h)	Conversion[b] (%)	M_w[c] (g.mol^{-1})	M_n[c] (g.mol^{-1})	$Đ$[c]
**1**	Moc_2O	0.1	1	97	34,500	15,000	2.3
**2**	Boc_2O	0.1	1	0	-	-	-
**3**	Boc_2O	0.1	78	0	-	-	-
**4**	Moc_2O	1	1	90	13,300	7,000	1.9
**5**	Boc_2O	1	1	25	-	-	-
**6**	Boc_2O	1	20	>99	18,400	10,200	1.8

[a] [dicarbonate]/[diacid] = 1.25, [diacid]/[(TMP)MgCl·LiCl] = 10,000, THF, 40 °C. [b] Conversion of carboxylic acid functions into anhydride linkages, as determined by ^{1}H NMR spectroscopy. [c] Determined by SEC.

Table S3. Influence of the solvent on the magnesium-catalyzed polycondensation of eicos-10-enedioic acid[a]

Entry	Solvent	Conversion[b] (%)	M_w[c] (g.mol^{-1})	M_n[c] (g.mol^{-1})	$Đ$[c]
1	THF	79	15,600	8,200	1.9
2	DMF[d]	91	54,600	26,000	2.1
3	Methyl-THF	77	15,000	7,900	1.9
4	Cyclohexanone	85	13,800	8,600	1.6

[a] General conditions: [eicos-10-enedioic acid]=[Moc_2O]=0.1 M, [Monomer]/[(TMP)MgCl·LiCl]=10,000, 1 h, 40°C. [b] As determined by ^{1}H NMR spectroscopy. [c] Determined by SEC. [d] The crude reaction mixture was not fully soluble during the purification procedure; the reported SEC result should therefore be taken with caution.

Orthogonal tandem synthesis of poly(eicos-10-ene-1,20-dicarboxylic anhydride)

In the glovebox, 10-undecenoic acid (965 mg, 5.245 mmol, 500 eq.), Zhan-1B (7.7 mg, 10.5 µmol, 1 eq.), (TMP)MgCl·LiCl (26.2 µL of a 0.010 M solution, 0.262 µmol, 0.025 eq. relative to Ru), and DMC (5.25 mL) were introduced into a Schlenk tube. The tube was removed from the glovebox, and acetic acid (3 µL, 52.4 µmol, 5 eq. relative to Ru) was added under argon.

The reaction mixture was heated at reflux for 30 min. Conversion of 10-undecenoic acid and olefin isomerization were evaluated by ^{1}H NMR spectroscopy. DMC was then removed under reduced pressure without isolation of the diacid intermediate. THF (5.25 mL) and dimethyl dicarbonate (0.35 mL, 3.28 mmol, 1.25 equiv. relative to the theoretical amount of diacid formed by self-metathesis) were added under argon. The mixture was stirred at 40 °C for 1 h. Volatile components were removed under reduced pressure, and the crude polymer was dissolved in dichloromethane and passed through a short plug of silica. Removal of the solvent afforded the target polyanhydride.

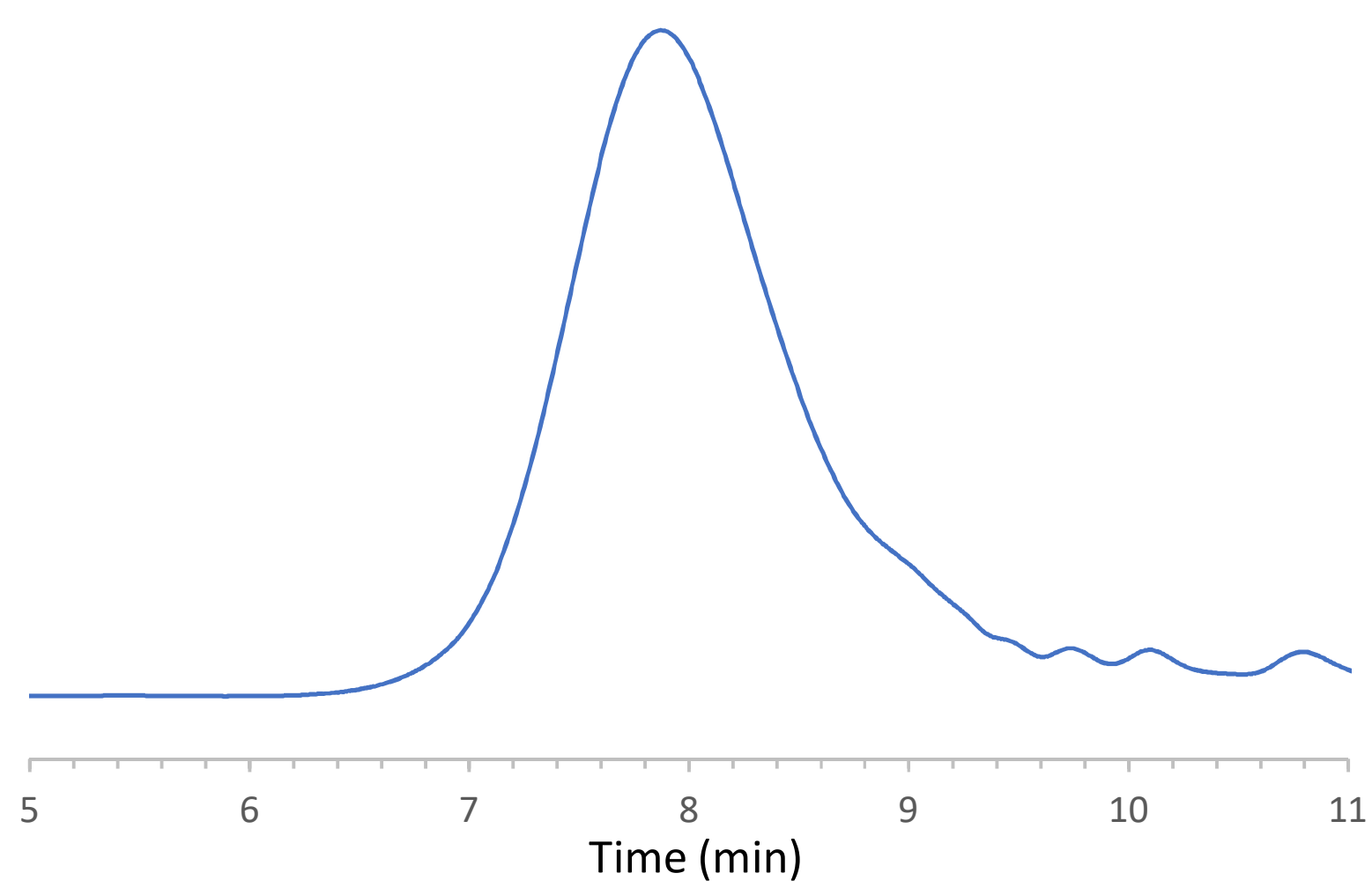

Figure S8. SEC chromatogram of the polyanhydride obtained by the orthogonal Ru/Mg tandem sequence (M_w = 30.8 kg mol^{-1}, Đ = 2.0).

Auto-tandem synthesis of poly(eicos-10-ene-1,20-dicarboxylic anhydride)

In a glovebox, 10-undecenoic acid (965 mg, 5.245 mmol, 500 eq.), Zhan-1B (7.7 mg, 10.5 µmol, 1 eq.), and DMC (5.25 mL) were introduced into a Schlenk tube. The tube was removed from the glovebox, and acetic acid (3 µL, 52.4 µmol, 5 eq. relative to Ru) was added under argon. The reaction mixture was heated at reflux for 30 min. Conversion of 10-undecenoic acid and olefin isomerization were evaluated by ^{1}H NMR spectroscopy. DMC was removed under

reduced pressure without isolation of the diacid intermediate. THF (5.25 mL) and dimethyl dicarbonate (0.35 mL, 3.28 mmol, 1.25 eq. relative to the theoretical amount of diacid) were then added under argon. The reaction mixture was stirred at 40 °C for 2 h. Volatile components were removed under reduced pressure, and the crude polymer was dissolved in dichloromethane and passed through a short plug of silica. Removal of the solvent afforded the target polyanhydride.

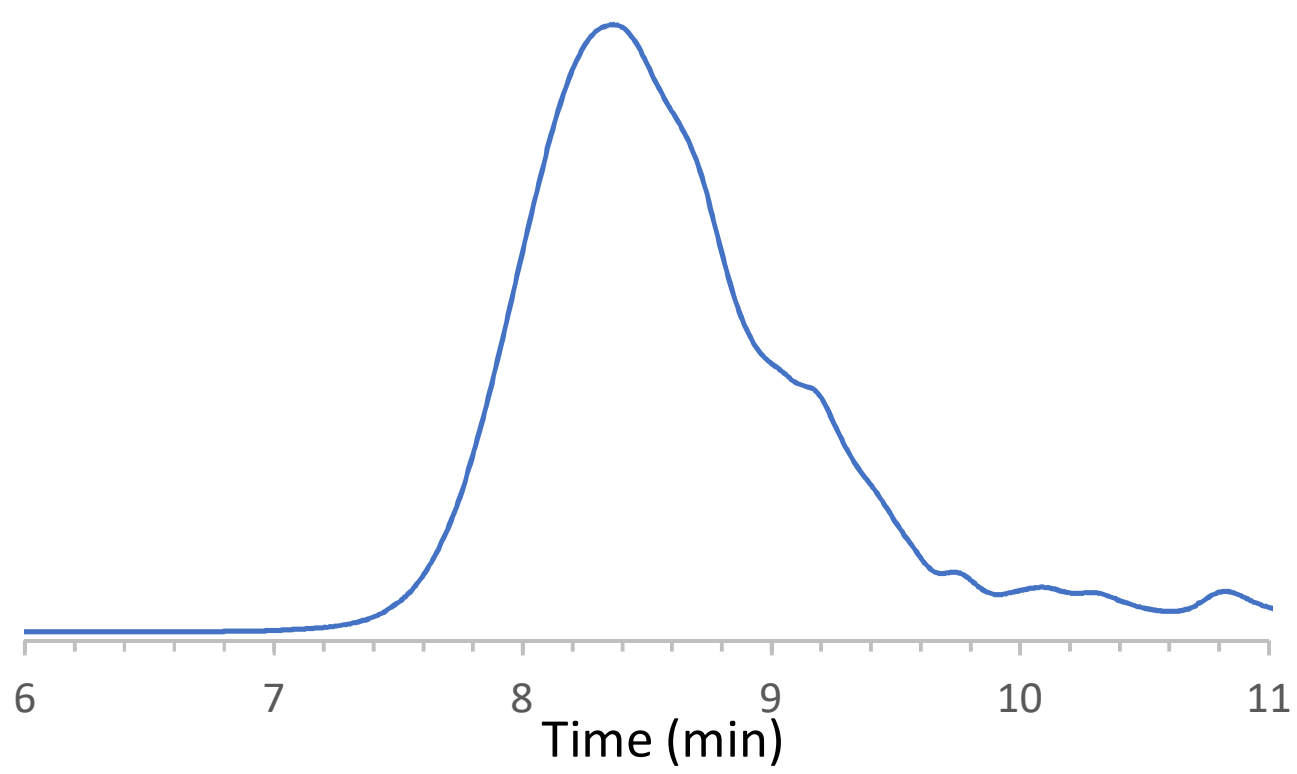

Figure S9. SEC chromatogram of the polyanhydride obtained by the auto-tandem sequence using Zhan-1B (M_w = 11.8 kg mol−1, $Đ$ = 1.6).

Formation of and investigations on a representative polyanhydride film

Polyanhydride was prepared from 170.3 mg (0.5 mmol, 1000 eq) eicos-10-ene dioic acid, 136.4 mg (0.625 mmol, 1250 eq) di-*tert*-butyl dicarbonate and 0.5 µmol (1 eq) of 2,2,6,6-tetramethylpiperidinylmagnesium chloride lithium chloride complex, in THF (1 mL), at 40°C with magnetic stirring at 700 rpm, and degassing by argon flow every 30 minutes. After 1h 45 min, the reaction was cooled down to RT and volatiles removed in vacuo. The resulting polymer was dissolved in DCM (1 ml), filtered through a silica short pad and thoroughly dried under vacuum to afford a white powder, which was stored under inert conditions. SEC revealed a M_w of 63.2 kg.mol^{-1} and $Đ$ of 3.8) (132 mg, isolated yield: 82%). The material was placed on a glass plate fitted with a home-made aluminium cast and heated at 70 °C for 30 minutes. A

second glass plate was put on the molten polyanhydride and pressure was applied using a 200 g weight for 30 minutes. Cooling down to room temperature and removal of the glass plates afforded a slightly transparent, off-white polyanhydride film.

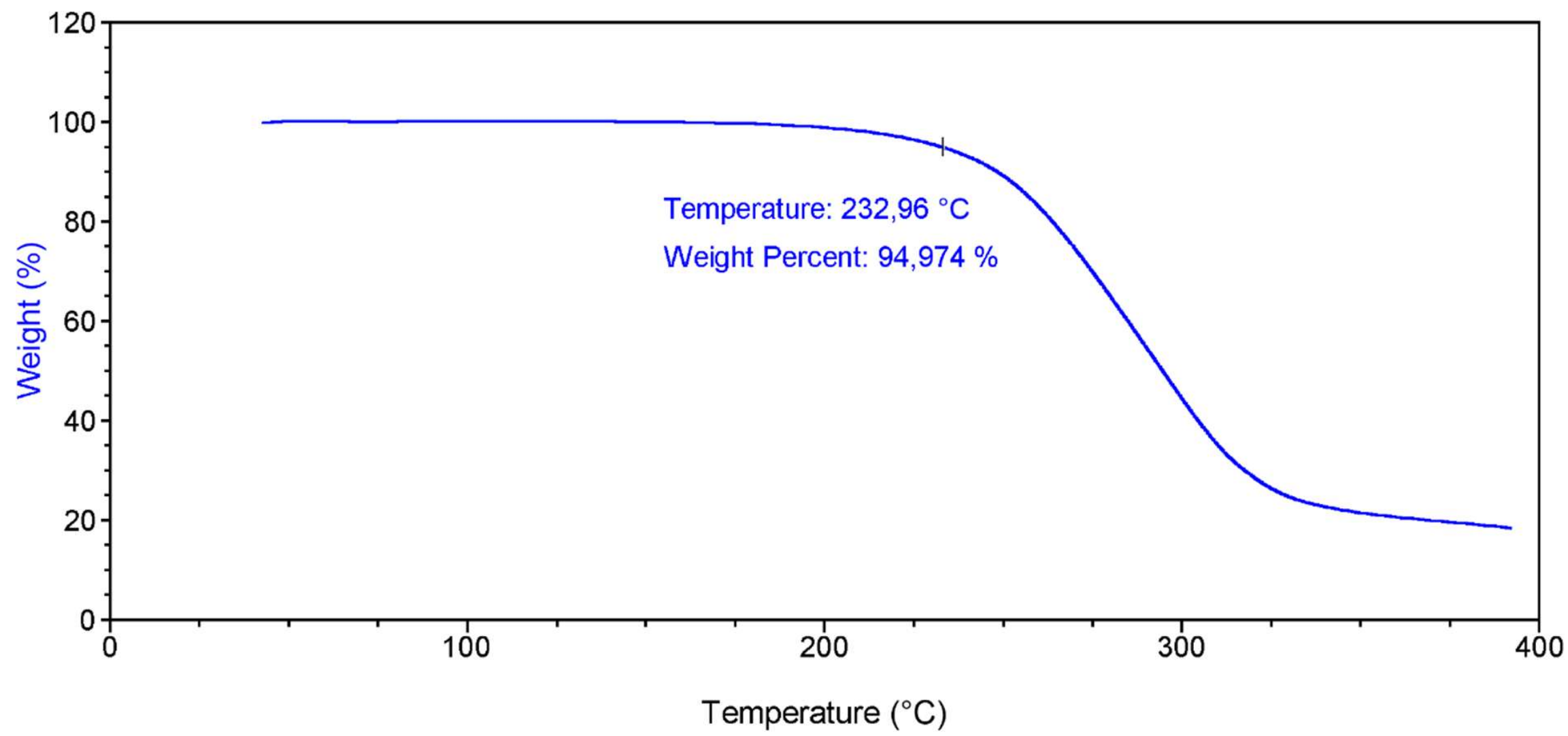

Figure S10. TGA trace of the polyanhydride film under nitrogen at a heating rate of 10 °C min^{-1}. The temperature corresponding to 5% mass loss was 233 °C.

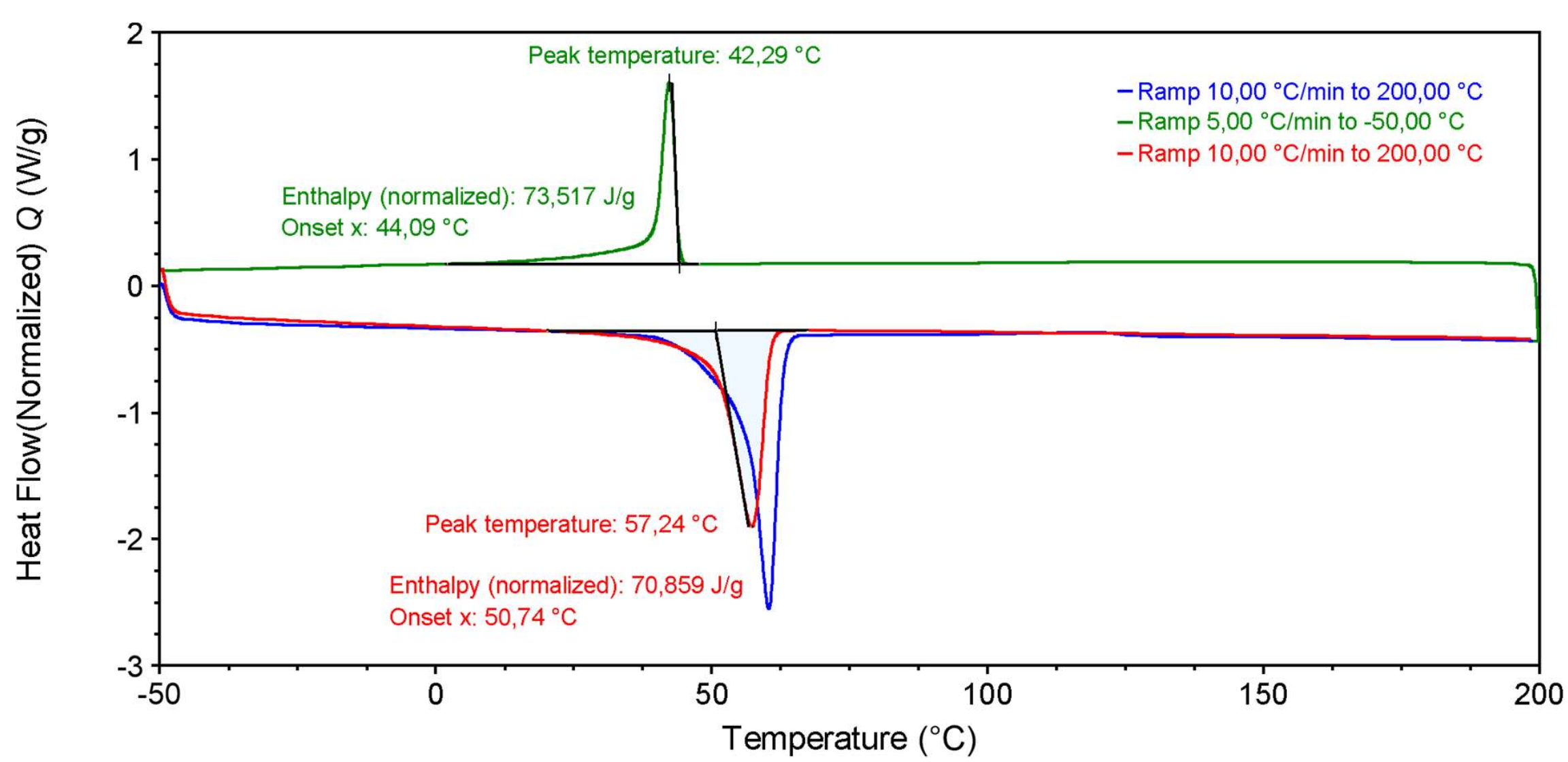

Figure S11. DSC heating and cooling traces of the polyanhydride film. The polymer displayed a melting transition at approximately 57 °C and a crystallization event at approximately 42 °C.

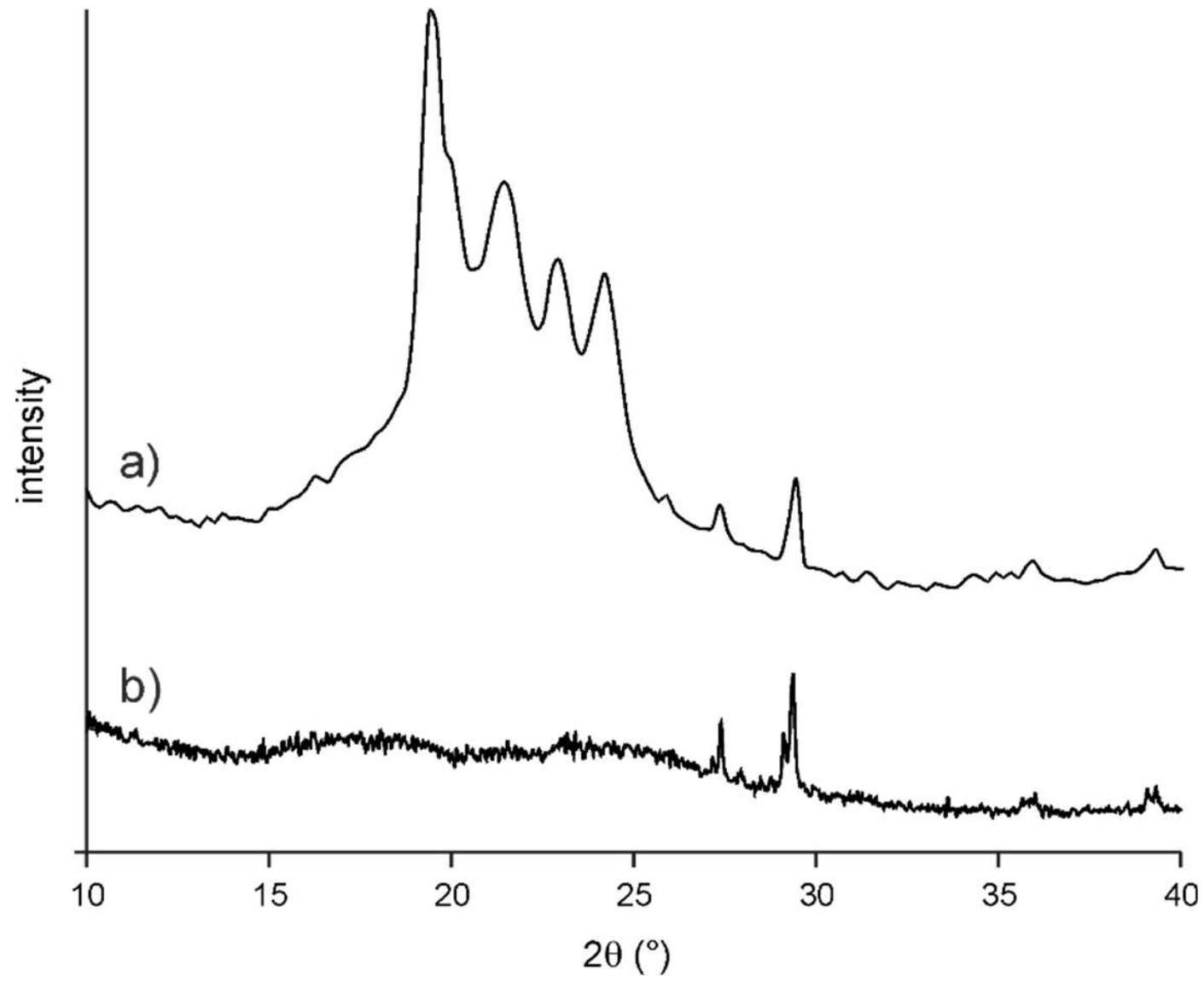

Figure S12. X-ray diffraction pattern of: a) the polyanhydride film and b) the empty sample holder

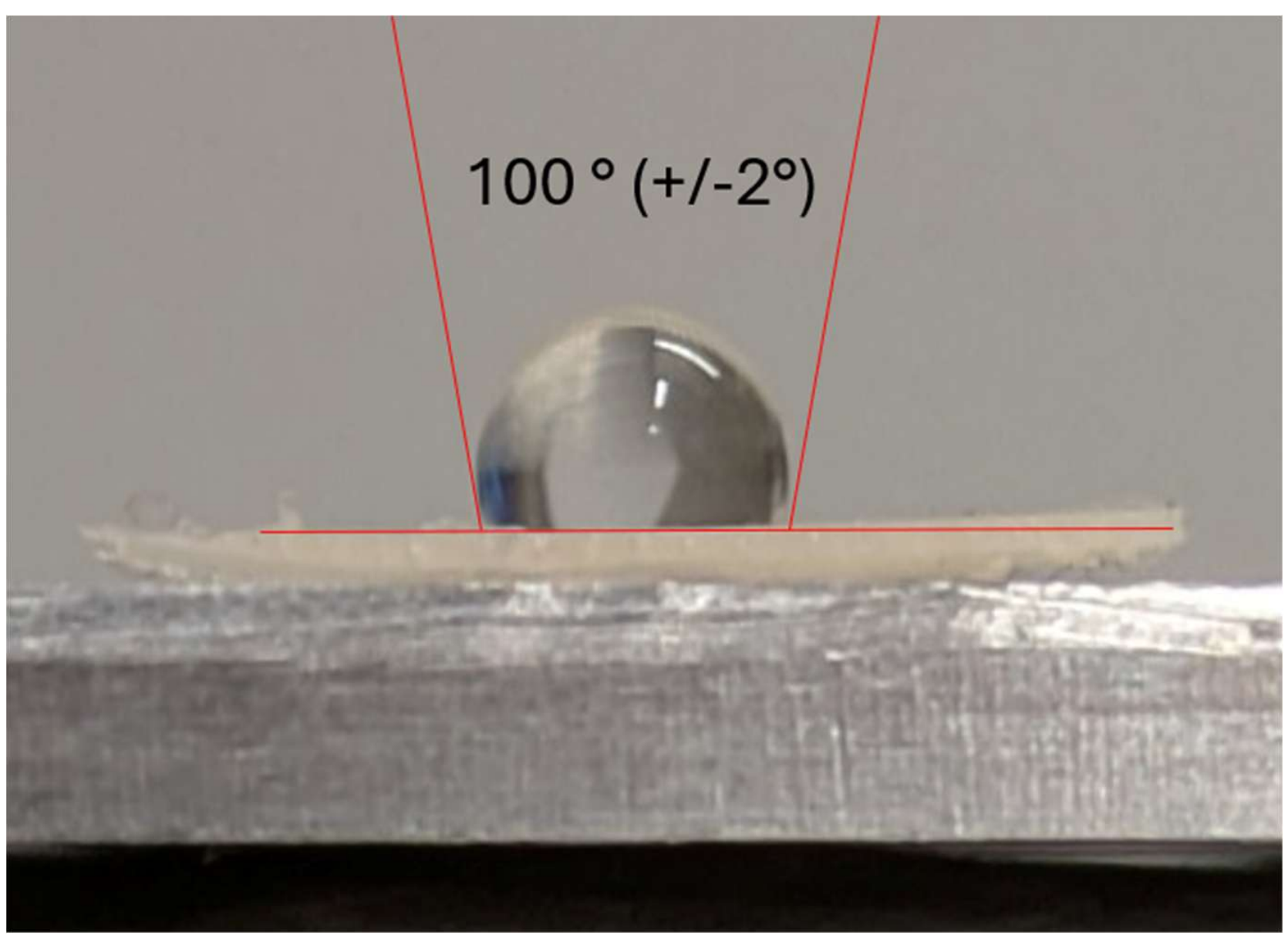

Figure S13. Representative side-view image of a water droplet deposited on a solvent-cast polyanhydride film during static water-contact-angle measurement
